# Sparse PLS discriminant analysis: biologically relevant feature selection and graphical displays for multiclass problems

**DOI:** 10.1186/1471-2105-12-253

**Published:** 2011-06-22

**Authors:** Kim-Anh Lê Cao, Simon Boitard, Philippe Besse

**Affiliations:** 1 Queensland Facility for Advanced Bioinformatics, University of Queensland, 4072 St Lucia, QLD, Australia; 2 UMR444 Laboratoire de Génétique Cellulaire, INRA, BP 52627, F-31326 Castanet Tolosan, France; 3 Institut de Mathématiques de Toulouse, Université de Toulouse et CNRS (UMR 5219), F-31062 Toulouse, France

## Abstract

**Background:**

Variable selection on high throughput biological data, such as gene expression or single nucleotide polymorphisms (SNPs), becomes inevitable to select relevant information and, therefore, to better characterize diseases or assess genetic structure. There are different ways to perform variable selection in large data sets. Statistical tests are commonly used to identify differentially expressed features for explanatory purposes, whereas Machine Learning wrapper approaches can be used for predictive purposes. In the case of multiple highly correlated variables, another option is to use multivariate exploratory approaches to give more insight into cell biology, biological pathways or complex traits.

**Results:**

A simple extension of a sparse PLS exploratory approach is proposed to perform variable selection in a multiclass classification framework.

**Conclusions:**

sPLS-DA has a classification performance similar to other wrapper or sparse discriminant analysis approaches on public microarray and SNP data sets. More importantly, sPLS-DA is clearly competitive in terms of computational efficiency and superior in terms of interpretability of the results via valuable graphical outputs. sPLS-DA is available in the R package mixOmics, which is dedicated to the analysis of large biological data sets.

## Background

High throughput technologies, such as microarrays or single nucleotide polymorphisms (SNPs) are seen as a great potential to gain new insights into cell biology, biological pathways or to assess population genetic structure. Microarray technique has been mostly used to further delineate cancers subgroups or to identify candidate genes for cancer prognosis and therapeutic targeting. To this aim, various classification techniques have been applied to analyze and understand gene expression data resulting from DNA microarrays ([1-3], to cite only a few). Genome wide association studies using SNPs aim to identify genetic variants related to complex traits. Thousands of SNPs are genotyped for a small number of phenotypes with genomic information, and clustering methods such as Bayesian cluster analysis and multidimensional scaling were previously applied to infer about population structure [4].

### Variable selection

As these high throughput data are characterized by thousands of variables (genes, SNPs) and a small number of samples (the microarrays or the patients), they often imply a high degree of multicollinearity, and, as a result, lead to severely ill-conditioned problems. In a supervised classification framework, one solution is to reduce the dimensionality of the data either by performing feature selection, or by introducing artificial variables that summarize most of the information. For this purpose, many approaches have been proposed in the microarray literature. Commonly used statistical tests such as t- or F-tests are often sensitive to highly correlated variables, which might be neglected in the variable selection. These tests may also discard variables that would be useful to distinguish classes that are difficult to classify [5]. Machine Learning approaches, such as Classification and Regression Trees (CART, [6]), Support Vector Machines (SVM, [7]) do not necessarily require variable selection for predictives purposes. However, in the case of highly dimensional data sets, the results are often difficult to interpret given the large number of variables. To circumvent this problem, several authors developed wrapper and embedded approaches for microarray data: Random Forests (RF, [8]), Recursive Feature Elimination (RFE, [3]), Nearest Shrunken Centroids (NSC, [9]), and more recently Optimal Feature Weighting (OFW, [5,10]). Other approaches were also used for exploratory purposes and to give more insight into biological studies. This is the case of Linear Discriminant Analysis (LDA), Principal Component Analysis (PCA, see [11,12] for a supervised version), Partial Least Squares Regression (PLS, [13], see also [14-16] for discrimination purposes), to explain most of the variance/covariance structure of the data using linear combinations of the original variables. LDA has often been shown to produce the best classification results. However, it has numerical limitations. In particular, for large data sets with too many correlated predictors, LDA uses too many parameters that are estimated with a high variance. There is therefore a need to either regularize LDA or introduce sparsity in LDA to obtain a parsimonious model. Another limitation of the approaches cited above is the lack of interpretability when dealing with a large number of variables.

Numerous *sparse *versions have therefore been proposed for feature selection purpose. They adapt well known ideas in the regression context by introducing penalties in the model. For example, a *l*_2 _norm penalty leads to Ridge regression [17] to regularize non invertible singular matrices. In particular, penalties of type *l*_1 _norm, also called Lasso [18], or *l*_0 _norm, were also proposed to perform feature selection, as well as a combination of *l*_1 _and *l*_2 _penalties [19]. These penalties (*l*_1 _and/or *l*_2_) were applied to the variable weight vectors in order to select the relevant variables in PCA [20,21] and more recently in Canonical Correlation Analysis [22-24] and in PLS [25-27]. [28,29] also proposed a penalized version of the PLS for binary classification problems. Recently, [30] extended the SPLS from [27] for multiclass classification problems and demonstrated that both SPLSDA and SPLS with an incorporated generalized framework (SGPLS) improved classification accuracy compared to classical PLS [31-33].

### Multiclass problems

In this study, we specifically focus on multiclass classification problems. Multiclass problems are commonly encountered in microarray studies, and have recently given rise to several contributions in the literature [34] and more recently [35,36]. Extending binary classification approaches to multiclass problems is not a trivial task. Some approaches can naturally extend to multiclass problems, this is the case of CART or LDA. Other approaches require the decomposition of the multiclass problem into several binary problems, or the definition of multiclass objective functions. This is the case, for example, of SVM one-vs.-one, one-vs.-rest or multiclass SVM.

### Sparse PLS-DA

We introduce a sparse version of the PLS for discrimination purposes (sPLS-Discriminant Analysis) which is a natural extension to the sPLS proposed by [25,26]. Although PLS is principally designed for regression problems, it performs well for classification problems [37,38]. Using this exploratory approach in a supervised classification context enables to check the generalization properties of the model and be assured that the selected variables can help predicting the outcome status of the patients. It is also important to check the stability of the selection, as proposed by [39,40]. We show that sPLS-DA has very satisfying predictive performances and is well able to select informative variables. In contrary to the two-stages approach recently proposed by [30], sPLS-DA performs variable selection and classification in a one step procedure. We also give a strong focus to graphical representations to aid the interpretation of the results. We show that the computational efficiency of sPLS-DA, combined with graphical outputs clearly give sPLS-DA a strong advantage to the other types of one step procedure variable selection approaches in the multiclass case.

### Outline of the paper

We will first discuss the number of dimensions to choose in sPLS-DA, and compare its classification performance with multivariate projection-based approaches: variants of sLDA [41], variants of SPLSDA and with SGPLS from [30]; and with five multiclass wrapper approaches (RFE, NSC, RF, OFW-cart, OFW-svm) on four public multiclass microarray data sets and one public SNP data set. All approaches perform internal variable selection and are compared based on their generalization performance and their computational time. We discuss the stability of the variable selection performed with sPLS-DA and the biological relevancy of the selected genes. Unlike the other projection-based sparse approaches tested, we show that sPLS-DA proposes valuable graphical outputs, also available from our R package mixOmics, to guide the interpretation of the results [42,43].

## Results and Discussion

In this section, we compare our proposed sPLS-DA approach with other sparse exploratory approaches such as two sparse Linear Discriminant Analyses (LDA) proposed by [41], and three other versions of sparse PLS from [30]. We also include in our comparisons several wrapper multiclass classification approaches. Comparisons are made on four public cancer microarray data sets and on one SNP data set. All these approaches perform variable selection in a supervised classification setting, i.e. we are looking for the genes/SNPs which can help classifying the different sample classes.

We first discuss the choice of the number of dimensions *H *to choose with sPLS-DA, the classification performance obtained with the tested approaches and the computational time required for the exploratory approaches. We then perform a stability analysis with sPLS-DA that can help tuning the number of variables to select and we illustrate some useful graphical outputs resulting from the by-products of sPLS-DA. We finally assess the biological relevancy of the list of genes obtained on one data set.

### Data sets

#### Leukemia

The 3-class Leukemia version [1] with 7129 genes compares the lymphocytes B and T in ALL (Acute Lymphoblastic Leukemia, 38 and 9 cases) and the AML class (Acute Myeloid Leukemia, 25 cases). The classes AML-B and AML-T are known to be biologically very similar, which adds some complexity in the data set.

#### SRBCT

The Small Round Blue-Cell Tumor Data of childhood (SRBCT, [44]) includes 4 different types of tumors with 23, 20, 12 and 8 microarrays per class and 2308 genes.

#### Brain

The Brain data set compares 5 embryonal tumors [45] with 5597 gene expression. Classes 1, 2 and 3 count 10 microarrays each, the remaining classes 4 and 8.

#### GCM

The Multiple Tumor data set initially compared 14 tumors [46] and 7129 gene expressions. We used the normalized data set from [47] with 11 types of tumor. The data set contains 90 samples coming from different tumor types: breast (7), central nervous system (12), colon (10), leukemia (29), lung (6), lymphoma (19), melanoma (5), mesotheolima (11), pancreas (7), renal (8) and uterus (9).

#### SNP data

The SNP data set considered in our study is a subsample of the data set studied by [48] in the context of the Human Genome Diversity Project, which was initiated for the purpose of assessing worldwide genetic diversity in human. The original data set of [48] included the genotypes at 525,910 single-nucleotide polymorphisms (SNPs) of 485 individuals from a worldwide sample of 29 populations. In order to work on a smaller sample size data set with still a large number of classes or populations (*K *= 7) and with a high complexity classification, we chose to keep only the African populations: Bantu Kenya, Bantu South Africa, Biaka Pygmy, Mandenka, Mbuty Pygmy, San and Yoruba. We filtered the SNPs with a Minor Allele Frequency> 0.05. For computational reasons, in particular to run the evaluation procedures using the wrapper methods, we randomly sampled 20,000 SNPs amongst the ones of the original dataset. The aim of this preliminary analysis is to show that sPLS-DA is well able to give satisfying results on biallelic discrete ordinal data (coded 0, 1 or 2, i.e. the number of mutant alleles at one SNP for one individual) compared to the other approaches.

### Choosing the number of sPLS-DA dimensions

In the case of LDA or sparse LDA (sLDA), it is of convention to choose the number of discriminant vectors *H *≤ min(*p*, *K *- 1), where p is the total number of variables and *K *is the number of classes. The *p*-dimensional data will be projected onto a *H*-dimensional space spanned by the first *H *discriminant vectors, also called *dimensions *in the case of sPLS.

**Figure 1:**
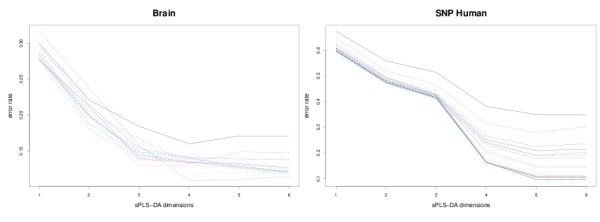
**Choosing the number of dimensions in sPLS-DA**. Estimated classification error rates for Brain and SNP (10 cross-validation averaged 10 times) with respect to each sPLS-DA dimension. The different lines represent the number of variables selected on each dimension (going from 5 to *p*).

To check if the same applies to sPLS-DA, we have plotted the mean classification error rate (10 cross-validation averaged 10 times) for each sPLS-DA dimension (Figure 1 for the Brain and SNP data sets, see Additional file 1 for the other data sets). We can observe that the estimated error rate is stabilized after the first *K *- 1 dimensions for any number of selected variables for the microarray data sets. For the SNP data set, *H *should be set to *K *- 2. The latter result is surprising, but can be explained by the high similarity between two of the classes: the Bantu Kenya and Banty South Africa populations, as illustrated later in the text.

Therefore, according to these graphics, reducing the subspace to the first *K *- 1 (*K *- 2) dimensions is sufficient to explain the covariance structure of the microarray (SNP) data. In the following, we only record the classification error rate obtained after *K *- 1 (*K *- 2) deflation steps have been performed with sPLS-DA - this also applies to the tested variants of SPLS from [30].

### Comparisons with other multiclass classification approaches

We compared the classification performance obtained with state-of-the-art classification approaches: RFE [49], NSC [9] and RF [8], as well as a recently proposed approach: OFW [10] that has been implemented with two types of classifiers, CART or SVM and has also been extended to the multiclass case [5]. These wrapper approaches include an internal variable selection procedure to perform variable selection.

We compared the classification performance of sPLS-DA to sLDA variants proposed by [41] based on a pooled centroids formulation of the LDA predictor function. The authors introduced feature selection by using correlation adjusted t-scores to deal with highly dimensional problems. Two shrinkage approaches were proposed, with the classical LDA (subsequently called sLDA) as well as with the diagonal discriminant analysis (sDDA). The reader can refer to [41] for more details and the associated R package sda.

Finally, we included the results obtained with 3 other versions of sparse PLS proposed by [30]. The SPLSDA formulation is very similar to what we propose in sPLS-DA, except that the variable selection and the classification is performed in two stages - whereas the prediction step in sPLS-DA is directly obtained from the by-products of the sPLS - see Section Methods. The authors in [30] therefore proposed to apply different classifiers once the variable selection is performed: Linear Discriminant Analysis (SPLSDA-LDA) or a logistic regression (SPLSDA-LOG). The authors also proposed a one-stage approach SGPLS by incorporating SPLS into a generalized linear model framework for a better sensitivity for multiclass classification. These approaches are implemented in the R package spls.

**Figure 2:**
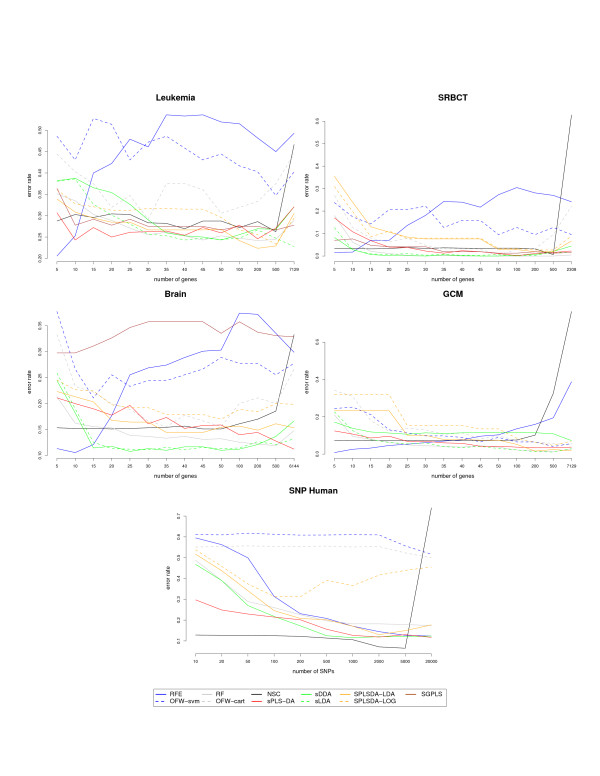
**Comparisons of the classification performance with other variable selection approaches**. Estimated classification error rates for Leukemia, SRBCT, Brain, GCM and the SNP data set (10 cross-validation averaged 10 times) with respect to the number of selected genes (from 5 to *p*) for the wrapper approaches and the sparse exploratory approaches.

Figure 2 displays the classification error rates estimated on each of the five data sets for all the tested approaches and Table 1 records the computational time required by the exploratory approaches to train the data on a given number of selected variables. Table 2 indicates the minimum estimated classification error rate obtained on each data set and for most of the approaches. Note that this table should be interpreted in conjunction with the results displayed in Figure 2 to obtain a better comprehensive understanding of how all approaches perform in relation with each other.

**Table 1:** Computational time

Data set	sDDA	sLDA	sPLS-DA	SPLS-LDA	SPLS-LOG	SGPLS
Leukemia	10	32	6	31	29	8

SRBCT	1	3	2	3	3	6

Brain	1	39	6	22	23	29

GCM	1	34	11	52	53	252

SNP	2	NA	17	749	731	NA

Computational time in seconds on a Intel(R) Core (TM) 2 Duo CPU 2.40 GHz machine with 4 GB of RAM to run the approaches on the training data for a chosen number of selected variables (50 for the microarray data and 200 for the SNP data).

**Table 2:** Minimum classification error rate estimated for each data set for the first best approaches (percentage) and the associated number of genes/SNPs that were selected.

Data set	rank 1	rank 2	rank 3	rank 4	rank 5	rank 6	rank 7	rank 8	rank 9
Leukemia	RFE	SPLSDA- LDA	LDA	SPLSDA- LOG	RF	DDA	sPLS	NSC	SGPLS
error rate	20.55	22.36	22.78	23.33	24.17	24.31	24.30	26.25	26.67
# genes	5	200	7129	500	200	50	10	500	500
SRBCT	RF	OFW- cart	DDA	LDA	sPLS	NSC	SGPLS	RFE	SPLSDA- LDA
error rate	0.00	0.00	0.00	0.00	0.16	0.63	1.27	1.58	1.90
# genes	30	50	30	100	100	500	50	5	200

Brain	RFE	DDA	LDA	sPLS	RF	SPLSDA- LDA	NSC	OFW- cart	SPLSDA- LOG
error rate	10.56	10.78	11.11	11.22	11.89	14.45	15.11	15.56	17.00
# genes	10	25	30	6144	500	35	20	35	50

GCM	RFE	LDA	RF	SGPLS- LDA	sPLS	OFW- svm	SGPLS- LOG	OFW- cart	NSC
error rate	0.81	1.14	1.22	1.63	3.41	4.01	4.71	4.88	7.23
# genes	5	500	500	200	200	500	500	7129	10

SNP	NSC	DDA	SPLS	RFE	SPLSDA- LDA	RF	SPLSDA- LOG	OFW- cart	OFW- svm
error rate	6.50	11.54	11.71	12.36	13.01	17.40	31.22	49.96	51.67
# SNPs	5000	1000	2000	20000	2000	20000	200	20000	20000

The approaches are ranked by their performances.

#### Details about the analysis

The aim of this section is to compare the classification performance of different types of variable selection approaches that may require some parameters to tune. We performed 10 fold cross-validation and averaged the obtained classification error rate accross 10 repetitions, and this for different variable selection sizes (Figure 2).

The wrapper approaches were run with the default parameters or the parameters proposed by the authors [8,50]. The sDDA and sLDA approaches are actually two-stages approaches as variables need to be ranked first before sLDA/DDA can be applied, but they do not require any other input parameter than the number of variables to select. sPLS-DA, SPLSDA-LOG/LDA and SGPLS require as input the number of PLS dimensions as discussed above. In addition, while sPLS-DA requires the number of variables to select on each dimension as an input parameter, SPLSDA-LOG/LDA and SGPLS require to tune the *η *parameter that varies between 0 and 1 - the closer to 1 the smaller variable selection size, so that it matched the variable selection sizes with the other approaches. SPLSDA-LOG/LDA are performed in two steps: one step for variable selection with SPLS and one step for classification.

#### Complexity of the data sets

All data sets differ in their complexity. For example, the 4-class SRBCT data set is known to be easy to classify [5] and most approaches - except NSC, have similar good performances. Analogously, the GCM data set that contains numerous classes (11) gives similar overall classification error rates for all approaches. The Brain and Leukemia data sets with 5 and 3 classes respectively seem to increase in complexity, and, therefore, lead to more accentuated discrepancies between the different approaches. The SNP data set is more complex due to the discrete ordinal nature of the data (3 possible values for each variable), a high number of populations (7) that have similar characteristics - some of them, for instance Bantu Kenya and Bantu South Africa, are closely related. Consequently, it can be expected that a large number of SNP may be needed to discriminate at best the different populations. This is what we observe, but, nonetheless, most approaches (except OFW) perform well, in particular NSC.

#### Computational efficiency

We only recorded the computational time of the exploratory approaches sDDA, sLDA, SPLSDA-LOG, SPLSDA-LDA, SGPLS and sPLS-DA as the wrapper approaches are computationally very greedy (the training could take from 15 min up to 1 h on these data). Some computation time could not be recorded as a R memory allocation problem was encountered (SNP data for sLDA and SGPLS).

The fastest approach is sDDA (except for Leukemia). This approach is not necessarily the one that performs the best, but is certainly the most efficient on large data sets. sPLS-DA is the second fastest one. The SPLSDA approaches were efficient on SRBCT but otherwise performed third, while SGPLS computation time was similar to sPLSDA except for large multiclass data set such as GCM.

#### Wrapper approaches

Amongst the wrapper approaches, RFE gave the best results for a very small selection of variables in most cases. The performance of RFE then dramatically decreased when the number of selected variables becomes large. This is due to the the backward elimination strategy adopted in the approach: the original variables are progressively discarded until only the 'dominant' mostly uncorrelated variables remain. RF seemed to give the second best performance for a larger number of variables. OFW-cart also performed well, as it aggregates CART classifiers, whereas OFW-svm performed rather poorly. This latter result might be due to the use of the one-vs-one multiclass SVM. NSC seemed affected by a too large number of variables, but performed surprisingly well on the SNP data.

#### sDDA/sLDA

Both variants gave similar results, but we could observe some differences in the GCM data set. In fact, [41] advised to apply sDDA for extremely high-dimensional data, but when a difference was observed between the two approaches (GCM, Leukemia), it seemed that sLDA performs the best. However, in terms of computational efficiency, sDDA was the most efficient.

#### SPLSDA-LOG/SPLSDA-LDA

SPLSDA-LDA gave better results than SPLSDA-LOG except for SRBCT where both variants performed similarly. On Leukemia, Brain and SNP, SPLSDA-LDA had a similar performance to sPLS-DA but only when the selection size became larger.

#### SGPLS

SGPLS performed similarly to sPLS-DA on SRBCT and gave similar performance to sPLS-DA on Leukemia when the selection size was large. However, it performed poorly in Brain where the number of classes becomes large and very unbalanced. SGPLS could not be run on GCM data as while tuning the *η *parameter, the smallest variable selection size we could obtain was 100, which did not make SGPLS comparable to the other approaches. On the SNP data SGPLS encountered R memory allocation issues.

#### sPLS-DA

sPLS-DA gave similar results to sDDA and sLDA in the less complex data sets SRBCT and GCM. The performance obtained on Brain was quite poor, but results were very competitive in Leukemia for a number of selected genes varying between 5 and 30. Note that the number of selected variables is the total number of variables selected accross the *K *- 1(*K *- 2) chosen dimensions (SNP data). In overall, sPLS-DA gave better results than the wrapper approaches, and remained very competitive to the other exploratory approaches. One winning advantage of sPLS-DA is the graphical outputs that it can provide (see next Section), as well as its computational efficiency.

### Stability analysis of sPLS-DA

It is useful to assess how stable the variable selection is when the training set is perturbed, as recently proposed by [39,40]. For instance, the idea of bolasso [40] is to randomize the training set by drawing boostrap samples or drawing *n*/2 samples in the training set, where *n *is the total number of samples. The variable selection algorithm is then applied on each subsample with a fixed number of variables to select and the variables that are selected are then recorded [40]. proposed to keep in the selection only the variables that were selected in all subsamples, whereas [39] proposed to compute a relative selection frequency and keep the most stable variables in the selection.

We chose to illustrate the latter option as we believe that the stability frequency, or probability, gives a better understanding of the number of stable discriminative variables that are selected in sPLS-DA. The highly correlated variables will get a higher probability of being selected in each subsample, while the noisy variables will have a probability close to zero. This stability measure can also guide the user in the number of variables to choose on each sPLS-DA dimension. Once the number of variables to select has been chosen for the first dimension, the stability analysis should be run for the second dimension and so on. Note that [39] proposed an additional perturbation by introducing random weights in the Lasso coefficients, called *random lasso*. This latter approach could not, however, be directly applied in the sPLS-DA algorithm due to its iterative nature.

**Figure 3:**
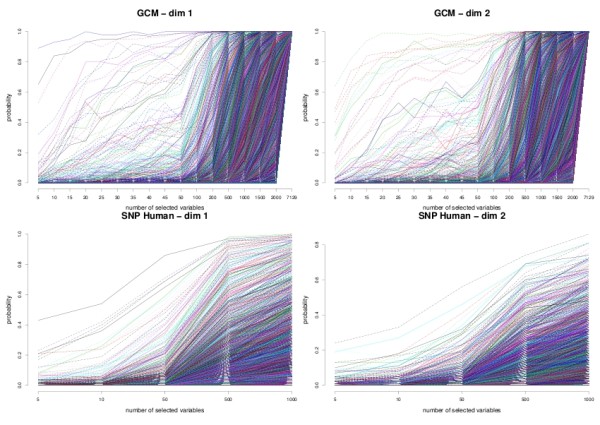
**Stability analysis**. Stability frequency using bolasso for the first two dimensions of sPLS-DA for GCM (top) and SNP data (bottom). One has to sequentially choose the most stabler genes/SNPs in the first dimension in order to pursue the stability analysis for the next sPLS-DA dimension.

Figure 3 illustrates the stability frequencies for the first two dimensions of the sPLS-DA for the GCM and SNP data sets using bootstrap sampling (i.e. of size *n*). The frequencies obtained on the GCM data set clearly show that the first 3 variables are often selected accross numerous bootstrap samples on the first dimension. We can see that while most microarray data could achieve a reasonably high stability frequency (see Additional file 2), this was not the case, however, for the SNP data. Several SNPs may contain similar information, this may induce a lower stability across the bootstrap samples for a small variable selection. Once the variable selection size grows larger, then there is enough stable information to be retained.

We also noticed that once we reached too many dimensions (i.e. close *K *- 1), then the frequencies of all variables dropped, which clearly showed that sPLS-DA could not distinguish between discriminative variables and noisy variables any more (not shown).

### Data visualization with sPLS-DA

#### Representing the samples and the variables

**Figure 4:**
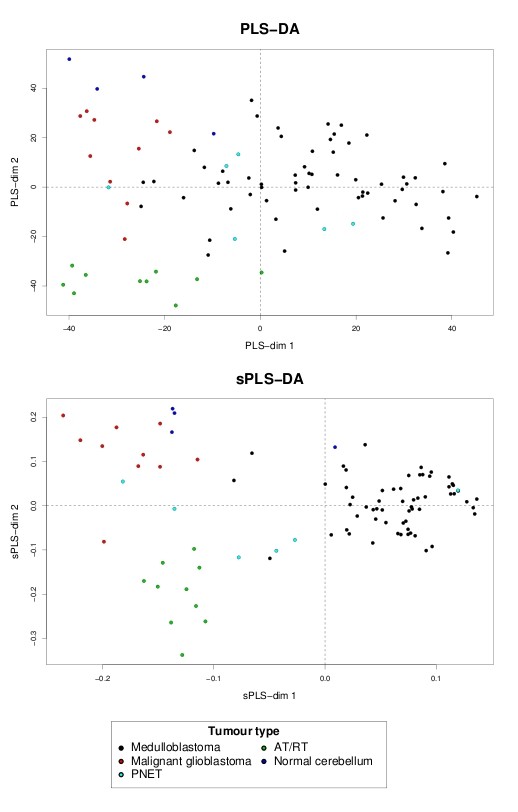
**Brain data: sample representation and comparison with classical PLS-DA**. Comparisons of the sample representation using the first 2 latent variables from PLS-DA (no variable selection) and sPLS-DA (26 genes selected).

**Figure 5:**
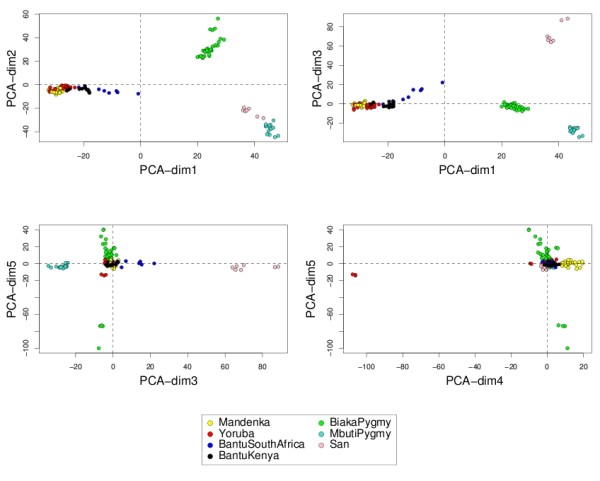
**SNP data: sample representation with PCA**. Sample representations using the first 5 principal components from PCA.

**Figure 6:**
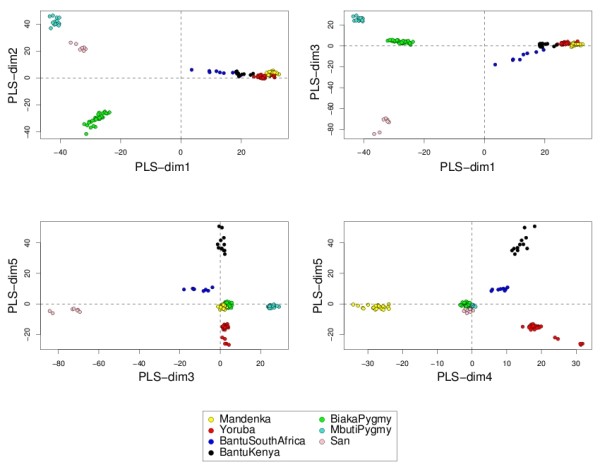
**SNP data: sample representation with classical PLS-DA**. Sample representation using the first 5 latent variables from PLS-DA (no SNPs selected).

**Figure 7:**
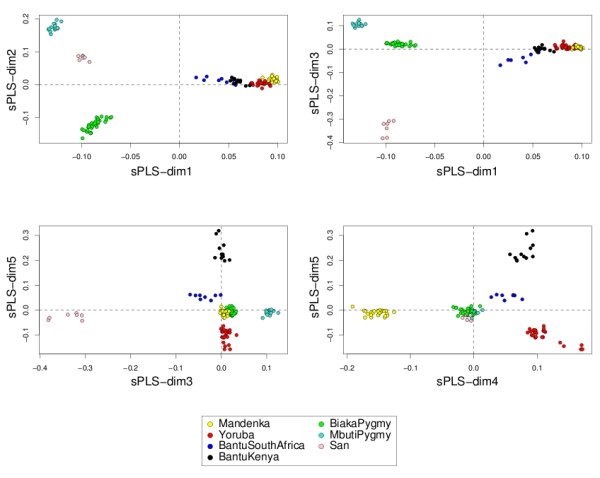
**SNP data: sample representation with sPLS-DA**. Sample representation using the first 5 latent variables from sPLS-DA (1000 SNPs selected on each dimension).

**Figure 8:**
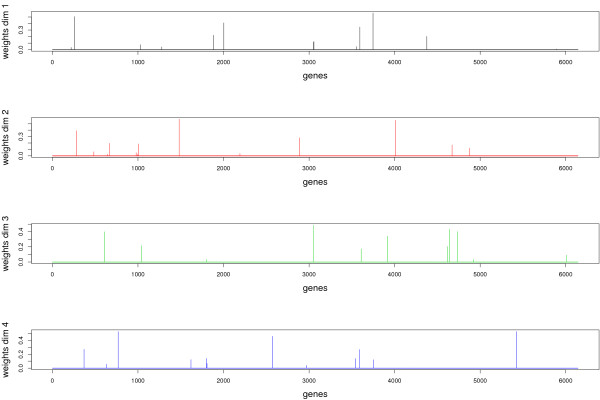
**Brain data: representation of the loading vectors**. Absolute value of the weights in the loading vectors for each sPLS-DA dimension. Only the genes with non zero weights are considered in the sPLS-DA analysis and are included in the gene selection.

**Figure 9:**
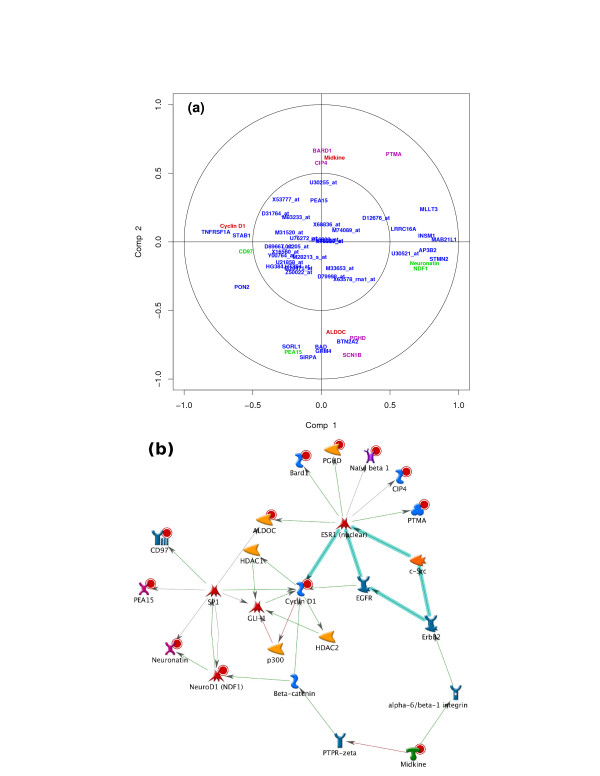
**Brain data: variable representation**. **(a) **projection of the sPLS-DA selected variables on correlation circles with the R mixOmics package; **(b) **biological network generated with GeneGo using the same list of genes. Genes that are present in the network **(b) **are labelled in green, red and magenta in **(a)**.

Data interpretation is crucial for a better understanding of highly dimensional biological data sets. Data visualization is one of the clear advantage of projection-based methods, such a Principal Component Analysis (PCA), the original PLS-DA or sPLS-DA, compared to the other tested approaches (wrapper methods, SPLSDA and SGPLS). The decomposition of the data matrix into loading vectors and latent variables provide valuable graphical outputs to easily visualize the results. For example, the latent variables can be used to represent the similarities and dissimilarities between the samples: Figure 4 illustrates the difference in the sample representation between classical PLS-DA (no variable selection) and sPLS-DA (26 genes selected on the first 2 dimensions) for the Brain data set. Variable selection for highly dimensional data sets can be beneficial to remove the noise and improve the samples clustering. A 3D graphical representation can be found in Additional file 3 with sPLS-DA. Figures 5, 6 and 7 compare the sample representation on the SNP data set using PCA (SNP data set only), classical PLS-DA and sPLS-DA on several principal components or PLS dimensions. On the full data set, PCA is able to discriminate the African hunter gatherers populations San, Mbuti and Biaka from the 4 other populations that are very similar (Mandeka, Yoruba, Bantu South Africa and Bantu Kenya). This is a fact that was previously observed [48] and it indicates a good quality of the data. With PCA however, the differentiation between the 4 populations Mandeka, Yoruba, Bantu South Africa and Bantu Kenya is not very clear, even for further dimensions (Figure 5). On the contrary to PCA, PLS-DA (Figure 6) and sPLS-DA (Figure 7) are able to discriminate further these 4 populations on dimensions 4 and 5. In particular, the Mandeka population is well differentiated on dimension 4, and so is the Yaruba population on dimension 5. In terms of sample representation and in contrary to what was obtained with the Brain data set (Figure 4), the difference between PLS-DA and sPLS-DA is not striking on this particular data set. This is probably because the SNP variables, although containing redundant information, are all informative and mostly not noisy. This also explains the good population clusters obtained with PCA (Figure 5). However, the variable selection performed in sPLS-DA has two advantages: firstly it reduces the size of the data set for further investigation and analyses; secondly, since each (s)PLS dimension focuses on the differentiation of some particular populations (Figures 5 and 6) and since sPLS selects an associated subset of variables on each on these dimensions, each of these subsets of variables is well able to differentiate these particular populations. This variable selection therefore gives more insight into the data (see [25] for more details). Figure 8 illustrates the weights in absolute value of the sparse loading vectors for each sPLS-DA dimension in the Brain data set. Only the genes with a non-zero weight are considered in the sPLS-DA analysis and were included in the gene selection (50 genes in total for this example). Generally, the sparse loading vectors are orthogonal to each other, which permits to uniquely select genes across all dimensions. The latent variables can also be used to compute pairwise correlations between the genes to visualize them on correlation circles and better understand the correlation between the genes on each dimension (Figure 9(a)). Note that this type of output is commonly used for Canonical Correlation Analysis.

On the contrary, the pooled centroid formulation used in sDDA and sLDA do not provide such latent variables, and, therefore, lack of such useful outputs. The same can be said about the wrapper approaches, which often have a much higher computational cost than the sparse exploratory approaches applied in this study.

### Brain data set: biological interpretation

#### Comparisons between the gene lists

The ultimate aim when performing variable selection is to investigate whether the selected genes (or SNPS) have a biological meaning. We saw for example that some of the tested approaches gave similar performances, even though they select different variables.

We therefore compared the lists of 50 genes selected with the different approaches on the Brain data set. Note that the selection size has to be large enough to extract known biological information from manually curated databases.

Unsurprisingly, given the variety of approaches used, there were not many genes in common: there were between 12 and 30 genes shared between sPLS-DA, sDDA, sLDA and SPLDA - sDDA and sLDA shared the most important number of genes (30). The gene selection from SGPLS grandly differed from the other multivariate approaches (between 2 and 9 genes). This may explain why the performance of SGPLS was pretty poor compared to the other approaches on the Brain data set. RF seemed to be the approach that selected the highest number of genes in common with all approaches except with NSC (between 10 and 23 genes). A fact to be expected was that there were very few commonly selected genes between the exploratory approaches and the wrapper approaches (between 2 and 10 genes).

We then investigated further the biological meaning of the selected genes. This analysis was performed with the GeneGo software [4] that outputs process networks, gene ontology processes as well as the list of diseases potentially linked with the selected genes.

It was interesting to see that in all these gene lists (except NSC and RFE), between 3 to 5 genes were linked to networks involved in neurogenesis, apoptosis, as well as DNA damage (sPLS-DA, sDDA) and neurophysiological processes (OFW-cart). Most of the lists that were selected with the wrapper approaches generated interesting gene ontology processes, such as degeneration of neurons (RF), synaptic transmission or generation of neurons (OFW-svm). On the contrary, the sparse exploratory approaches seemed to pinpoint potential biomarkers linked with relevant diseases: central nervous system and brain tumor (sPLS-DA), Sturge Weber syndrome, angiomatosis, brain stem (sDDA, sLDA), neurocutaneous syndrome (sDDA), neurologic manifestations and cognition disorders (SGPLS).

This preliminary analysis shows that the different approaches are able to select relevant genes linked to the biological study and are able to select complementary information. This was also the conclusion drawn in [10].

#### Further biological interpretation with the sPLS-DA list

Using the GeneGo software, known biological networks were generated from the list of genes selected with sPLS-DA - 26 genes in total for the first two dimensions. For example, the network represented in Figure 9(b) is based on 12 of these selected genes (indicated with a red dot), which are involved in biological functions such as cell differentiation, cellular developmental process and central nervous system development. These genes are organised around two transcription factors, ESR1 and SP1. SP1 can activate or repress transcription in response to physiological and pathological stimuli and regulates the expression of a large number of genes involved in a variety of processes such as cell growth, apoptosis, differentiation and immune responses.

Interestingly, all 12 genes present in the network were also found to be highly correlated to the sPLS-DA dimensions 1 and 2 (indicated in green for the ESR1 network, magenta for the SP1 network and red for common genes in both subgraphs). This latter result suggests a. that the first (second) dimension of sPLS-DA seems to focus on the SP1 (ESR1) network and b. that the genes selected with sPLS-DA are of biological relevance (see Table 3 for a description of most genes). Further investigation would be required to give more insight into the sPLS-DA gene selection.

**Table 3:** Brain data: Biological relevance of some of the selected genes

Bard1	Plays a central role in the control of the cell cycle in response to DNA damage
PGDH	Possibly involved in development and maintenance of the blood-brain, blood-retina, blood-aqueous humor and blood-testis barrier. It is likely to play important roles in both maturation and maintenance of the central nervous system and male reproductive system
Na(v) Beta1	Involved in the generation and propagation of action potentials in muscle and neuronal cells
NDF1	Differentiation factor required for dendrite morphogenesis and maintenance in the cere bellar cortex
Neuronatin	May participate in the maintenance of segment identity in the hindbrain and pituitary development, and maturation or maintenance of the overall structure of the nervous system
PEA15	death effector domain (DED)-containing protein predominantly expressed in the central nervous system, particularly in astrocytes
CD97	Receptor potentially involved in both adhesion and signalling processes early after leukocyte activation. Plays an essential role in leukocyte migration
ALDOC	is expressed specifically in the hippocampus and Purkinje cells of the brain
Cyclin D1	The protein encoded by this gene has been shown to interact with tumor suppressor protein Rb Mutations, amplification and overexpression of this gene, which alters cell cycle progression, are observed frequently in a variety of tumors and may contribute to tumour genesis

Description of the genes or proteins encoded by the genes selected by sPLS-DA and present in the known GeneGO biological network.

## Conclusions

In this article, we showed that sPLS could be naturally extended to sPLS-DA for discrimination purposes by coding the response matrix *Y *with dummy variables. sPLS-DA often gave similar classification performance to competitive sparse LDA approaches in multiclass problems. Undoubtedly, the sparse approaches that we tested are extremely competitive to the wrapper methods, which are often considered as black boxes with no intuitive tuning parameters (such as the kernels to use in the SVM). The preliminary biological analysis showed that some tested approaches brought relevant biological information. The PLS-based approaches such as the sPLS-DA approach that we propose have a well established framework for class prediction. The computational efficiency of sPLS-DA as well as the valuable graphical outputs that provide easier interpretation of the results make sPLS-DA a great alternative to other types of variable selection techniques in a supervised classification framework. We also showed that a stability analysis could guide the parameter tunings of sPLS-DA. On the Brain data set, we showed that sPLS-DA selected relevant genes that shed more light on the biological study. For these reasons, we believe that sPLS-DA provides an interesting and worthwhile alternative for feature selection in multiclass problems.

## Methods

In this section, we introduce the sparse Partial Least Squares Discriminant Analysis (sPLS-DA) to perform feature selection. sPLS-DA is based on Partial Least Squares regression (PLS) for discrimination analysis, but a Lasso penalization has been added to select variables. We denote *X *the *n *× *p *sample data matrix, where *n *is the number of patients or samples, and *p *is the number of variables (genes, SNPs, ...). In this supervised classification framework, we will assume that the samples *n *are partitioned into *K *groups.

### Introduction on PLS Discriminant Analysis

Although Partial Least Squares [13] was not originally designed for classification and discrimination problems, it has often been used for that purpose [38,51]. The response matrix *Y *is qualitative and is recoded as a dummy block matrix that records the membership of each observation, i.e. each of the response categories are coded via an indicator variable. The PLS regression (now PLS-DA) is then run as if *Y *was a continuous matrix. Note that this might be wrong from a theoretical point of view, however, it has been previously shown that this works well in practice and many authors have used dummy matrice in PLS for classification [30,37,51,52].

PLS constructs a set of orthogonal components that maximize the sample covariance between the response and the linear combination of the predictor variables. The objective function to be solved can be written as

where *u_h _*and *v_h _*are the *h*th left and right singular vector of the singular value decomposition (SVD) of *X^T ^Y *respectively [53] for each iteration or dimension *h *of the PLS. These singular vectors are also called loading vectors and are associated to the *X *and *Y *data set respectively.

In the case of discrimination problems, the PLS model can be formulated as follows:

where *β *is the matrix of the regression coefficients and *E *is the residual matrix. To give more details, *β *= W*V ^T^, where V is the matrix containing the loading vectors (or right singular vectors from the SVD decomposition) (*v*_1_, ..., *v_H _*) in columns, W* = *W *(*U^T ^W *)^-1^, where *W *is the matrix containing the regression coefficients of the regression of *X *on the latent variable , and *U *is the matrix containing the loading vectors (or left singular vectors from the SVD decomposition) (*u*_1_, ..., *u_H _*) in columns. More details about the PLS algorithm and the PLS model can be found in the reviews of [53,54]. The prediction of a new set of samples is then

The identity of the class membership of each new sample (each row in *Y_new _*) is assigned as the column index of the element with the largest predicted value in this row.

#### Discriminant PLS for large data sets

Numerous variants of PLS-DA have been proposed in the literature to be adapted to classification problems for large data sets such as microarray. Iterative Reweighted PLS was first proposed by [31] to extend PLS into the framework of generalized linear models. In the same context, [51,55,56] proposed a two-stage approach, first by extracting the PLS-DA latent variables to reduce the dimension of the data, and then by applying logistic discrimination or polychotomous discrimination in the case of multiclass problems. To avoid infinite parameters estimates and non convergence problems, other authors [32] extended the work of [31] by applying Firth's procedure to avoid (quasi) separation, whereas [33] combined PLS with logistic regression penalized with a ridge parameter. The response variables *Y *is modelled either as a dummy matrix [51,55,56], or as a pseudo-response variable whose expected value has a linear relationship with the covariates [33]. The approach proposed by [32] updates the adjusted dependent variable as the response rather than working with the original outcome. While these authors propose to address the problem of dimension reduction, they still require to perform gene filtering beforehand, with, for example, *t*-statistics or other filtering criterion such as the BSS/WSS originally proposed by [2].

### sparse PLS Discriminant Analysis

#### sparse PLS for two data sets

The sparse PLS proposed by [25,26] was initially designed to identify subsets of correlated variables of two different types coming from two different data sets *X *and *Y *of sizes (*n *× *p*) and (*n *× *q*) respectively. The original approach was based on Singular Value Decomposition (SVD) of the cross product . We denote *u_h _*(*v_h_*) the left (right) singular vector from the SVD, for iteration *h*, *h *= 1 ... *H *where *H *is the number of performed deflations - also called chosen *dimensions *of the PLS. These singular vectors are named *loading vectors *in the PLS context. Sparse loading vectors were then obtained by applying *l*_1 _penalization on both *u_h _*and *v_h_*. The optimization problem of the sPLS minimizes the Frobenius norm between the current cross product matrix and the loading vectors:(1)

where *P*_λ1 _(*u_h_*) = *sign*(*u_h_*)(|*u_h_*| - λ_1_)_+_, and *P*_λ2 _(*v_h_*) = *sign*(*v_h_*)(|*v_h_*| - λ_2_)_+ _are applied componentwise in the vectors *u_h _*and *v_h _*and are the soft thresholding functions that approximate Lasso penalty functions [21]. They are simultaneously applied on both loading vectors. The problem (1) is solved with an iterative algorithm and the *X_h _*and *Y_h _*matrices are subsequently deflated for each iteration *h *(see [25] for more details). For practical purposes, sPLS has been implemented in the R package mixOmics such that the user can input the number of variables to select on each data set rather than the penalization parameters *λ*_1 _and *λ*_2_.

#### sPLS extended to sPLS-DA

The extension of sparse PLS to a supervised classification framework is straightforward. The response matrix *Y *of size (*n *× *K*) is coded with dummy variables to indicate the class membership of each sample. Note that in this specific framework, we will *only perform variable selection on the X data set*, i.e., we want to select the discriminative features that can help predicting the classes of the samples. The *Y *dummy matrix remains unchanged. Therefore, we set  and the optimization problem of the sPLS-DA can be written as:

with the same notation as in sPLS. Therefore, the penalization parameter to tune is λ. Our algorithm has been implemented to choose the number of variables to select rather than λ for practical reasons. For the class prediction of test samples, we use the *maximum *distance as presented above for the PLS case as it seemed to be the one that worked better in practice for multiclass problems. Note that other distances such as the centroids or Malhanobis distances are also implemented in the mixOmics package [42,43]. In the results section, we illustrated how to tune the PLS dimension *H *as well as the number of *X *variables to select.

#### sPLS-DA for multiclass classification

In binary problems, sPLS-DA was shown to bring relevant results in microarray cancer data sets (see [57]). In this paper, we investigated the use of sPLS-DA in the more complex multiclass case, as PLS-DA and sPLS-DA are naturally adapted to multiclass problems. In this paper, we did not attempt to address the specific problem of unbalanced classes, that would require the development of appropriately weighted multiclass objective functions for wrapper classification approaches (see for example [58]).

#### Parameters to tune in sPLS-DA

There are two parameters to tune in sPLS-DA: the number of dimensions *H*, and the number of variables to select on each dimension. In the Results Section, we showed that for most cases, the user could set *H *= *K *- 1, similar to what is advised in a LDA case. The number of variables to select is more challenging given the complexity of such data sets and is still as an open question. The tuning of such parameter can be guided through the estimation of the generalisation classification error and a stability analysis. However, these analyses might be seriously limited by the small number of samples. Most importantly, the user should keep in mind that a close interaction with the biologists is necessary to carefully tune this parameter in order to answer biological questions. Sometimes, an optimal but too short gene selection may not suffice to give a comprehensive biological interpretation, and experimental validation might be limited in the case of a too large gene selection.

## Competing interests

The authors declare that they have no competing interests.

## Authors' contributions

KALC performed the statistical analysis, wrote the R functions and drafted the manuscript. SB preprocessed the SNP data and helped to draft the manuscript. PB participated in the design of the manuscript and helped to draft the manuscript. All authors read and approved the final manuscript.

## Supplementary Material

Additional file 1 **Tuning the number of dimensions in sPLS-DA**. Estimated classification error rates for Leukemia, SRBCT and GCM (10 cross-validation averaged 10 times) with respect to each sPLS-DA dimension. The different lines represent the number of variables selected on each dimension (going from 5 to *p*). Click here for file

Additional file 2 **Stability analysis**. Stability frequency using bolasso for the first two dimensions of sPLS-DA for Brain (top) and SRBCT data (bottom). One has to sequentially choose the most stabler genes/SNP in the first dimension in order to go on to the next sPLS-DA dimension. Click here for file

Additional file 3 **Brain data: sample representation in 3D**. Example of 3D samples plot using the first 3 latent variables from sPLS-DA with the R mixOmics package. Click here for file
