# Supplementary figures and images for: Sparse PLS discriminant analysis: biologically relevant feature selection and graphical displays for multiclass problems
Source: BMC Bioinformatics. 2011 Jun 22;12:253. doi: 10.1186/1471-2105-12-253 (PMC3133555; doi:10.1186/1471-2105-12-253)

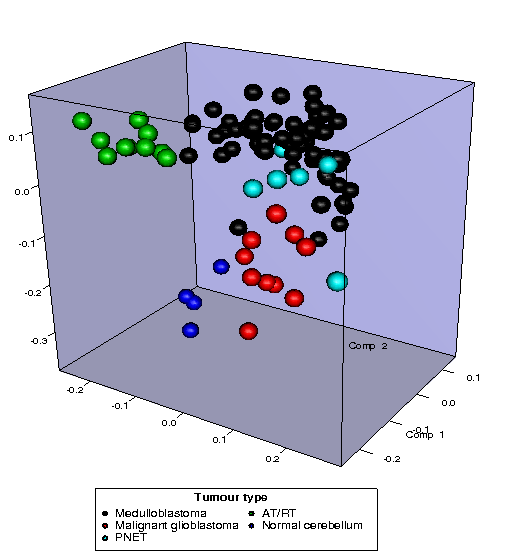

Supplement: Additional file 3 — Brain data: sample representation in 3D. Example of 3D samples plot using the first 3 latent variables from sPLS-DA with the R mixOmics package. [file 1471-2105-12-253-S3.PNG]
